# Augmented laminography, a correlative 3D imaging method for revealing the inner structure of compressed fossils

**DOI:** 10.1038/srep41413

**Published:** 2017-01-27

**Authors:** Marcus Zuber, Michael Laaß, Elias Hamann, Sophie Kretschmer, Norbert Hauschke, Thomas van de Kamp, Tilo Baumbach, Thomas Koenig

**Affiliations:** 1 Karlsruhe Institute of Technology (KIT), Institute for Photon Science and Synchrotron Radiation (IPS) & Institute for Beam Physics and Technology (IBPT), Hermann-von-Helmholtz-Platz 1, 76344 Eggenstein-Leopoldshafen, Germany; 2 Karlsruhe Institute of Technology (KIT), Laboratory for Applications of Synchrotron Radiation (LAS), Kaiserstr. 12, 76131 Karlsruhe, Germany; 3 University of Duisburg-Essen, Department of General Zoology, Faculty of Biology, Universitätsstr. 5, 45117 Essen, Germany; 4 Martin-Luther-University Halle-Wittenberg, Institute of Geosciences and Geography, Von-Seckendorff-Platz 3, 06120 Halle (Saale), Germany; 5 Ziehm Imaging GmbH, Donaustr. 31, 90451 Nuremberg, Germany

## Abstract

Non-destructive imaging techniques can be extremely useful tools for the investigation and the assessment of palaeontological objects, as mechanical preparation of rare and valuable fossils is precluded in most cases. However, palaeontologists are often faced with the problem of choosing a method among a wide range of available techniques. In this case study, we employ x-ray computed tomography (CT) and computed laminography (CL) to study the first fossil xiphosuran from the Muschelkalk (Middle Triassic) of the Netherlands. The fossil is embedded in micritic limestone, with the taxonomically important dorsal shield invisible, and only the outline of its ventral part traceable. We demonstrate the complementarity of CT and CL which offers an excellent option to visualize characteristic diagnostic features. We introduce *augmented laminography* to correlate complementary information of the two methods in Fourier space, allowing to combine their advantages and finally providing increased anatomical information about the fossil. This method of augmented laminography enabled us to identify the xiphosuran as a representative of the genus *Limulitella*.

## Overview

X-ray experiments are instrumental in assessing the features of a very wide range of objects studied in diverse fields, such as medical imaging, biology, materials science or industrial quality assurance tasks. Two dimensional (2D) projections are easily obtained, yet suffer from overlapping internal structures that are cast on the x-ray detector on top of each other. A method allowing to investigate an object’s interior structure is computed tomography (CT), which combines many projections acquired over an angular interval of at least 180° to determine the distribution of attenuation coefficients, representing the object’s ability to absorb x-rays^1^. While single projection radiographies are quickly obtained, performing a CT scan promises a wealth of three-dimensional (3D) information at the cost of longer acquisition times and radiation exposure.

Albeit being the most famous examples of x-ray imaging modalities, these two extremes – projection radiography and CT scans – do not represent the only options available. In particular, even if neither radiation dose nor acquisition times are of concern, CT may not be the optimal choice or even suitable at all to study a certain class of specimens. One reason for CT potentially failing to properly describe an object lies in x-ray penetration: If the object is too thick, it will not allow a sufficient amount of x-rays to pass, and no usable image will form on the detector. This problem of overly strong absorption is usually circumvented by choosing beam directions along such object axes that path lengths are shortest, which allows high quality CT images especially for objects with an approximately cylindrical symmetry, including human anatomy.

CT acquisition becomes more difficult when the object is only thin along one spatial dimension, i.e. if one deals with flat, but laterally extended specimens. In this case, projection images can already reveal some features, but 3D information may still be highly desirable. For such samples, laminographic techniques come into play. While they differ in implementation, a defining characteristic is that laminography attempts to recover some 3D information by investigating the object from different, but preferably such directions along which it is comparably thin and the transmittivity stays similar. Typically, laminography limits the achievable resolution along one spatial dimension, resulting in a characteristic and anisotropic blurring.

**Figure 1:**
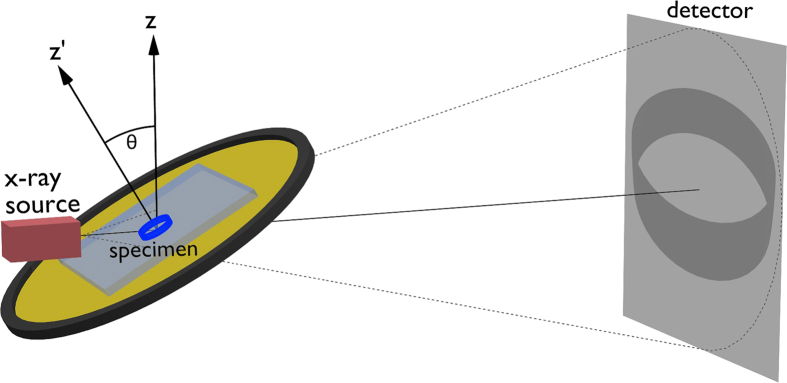
Scheme of a CL experiment, which aims at obtaining 3D information about a region-of-interest (blue boundary in the specimen, dark grey on the detector).

**Figure 2:**
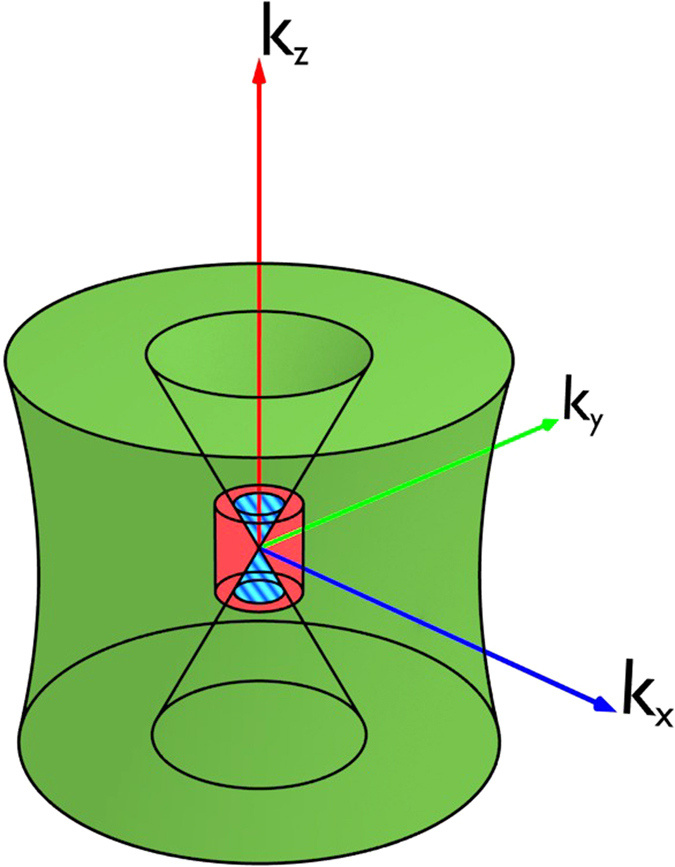
Sampled areas in the Fourier space of the reconstructed volume. The green volume found outside the two inner cones relates to a computed laminography and the red volume to a low resolution computed tomography scan. The blue stripes correspond to the domain where the missing information in the laminography data can be reconstructed from the CT scan.

In this work, we employ computed laminography (CL)^2^. It can be considered a generalization of CT through tilting the rotation axis against the central beam axis by the laminographic angle *θ*. As can be seen in Fig. 1, computed laminography substantially reduces the amount of material that needs to be penetrated by the x-ray beam. The effects of the tilted rotation axis on image quality are equivalent to those of circular tomosynthesis^2^,^3^,^4^,^5^. In a simplified view, a specimen’s lateral structures are mostly preserved, while coarse and vertical structures are blurred. Mathematically, choosing *θ* > 0 leads to two cones in the Fourier space of the object function that remain unsampled during image acquisition (Fig. 2). The larger *θ*, the more information is lost, and the more blurring occurs. Conventional (parallel beam) CT represents one limiting case of laminography for *θ* = 0, where these cones vanish and the complete Fourier space is sampled up to the Nyquist frequency. Laminography with *θ* = 90° corresponds to redundant sequences of rotated, yet ordinary radiography.

Another, less known advantage of CL is that of a potentially increased spatial resolution along those directions that do not suffer from any blurring. These correspond to spatial frequencies outside the missing cones. To illustrate this advantage of an increased resolution along some spatial directions, we briefly state two requirements for a CT acquisition to correctly reconstruct an image volume without prior knowledge. First, the projected region-of-interest (ROI) must never leave the detector while the specimen rotates. Second, none of the regions that leave the detector must overlap with that ROI during rotation. While the first condition is easily satisfied for both CT and CL, the second is more restrictive and requires reducing the geometric magnification in a CT experiment to make the object’s complete projection fit on the detector in the lateral direction, for any value of the rotation angle *φ*. Put differently, the resolution of the CT scan is limited by the requirement that the tomographic field-of-view (FOV) has to cover the full sample in the lateral direction to avoid truncation artifacts. Tilting the axis of rotation, however, allows fulfillment of the second condition in a CL experiment for larger magnifications than would be possible for conventional CT. Hence, the information loss around one direction due to missing information may be outweighed by an increase of spatial resolution along the others.

CL thus allows zooming into an ROI and so represents a case of interior tomography that is applicable to flat specimens. Such techniques have been presented in the past mostly for regular CT, each requiring some form of prior knowledge. This knowledge may be represented by assumptions on object composition or can be directly extracted from a low resolution scan that images the specimen as a whole. These methods usually work by forward projecting the low resolution CT scan to complete the missing parts of the high resolution ROI images^6^,^7^,^8^,^9^,^10^. The high resolution CT scan is generally performed by moving the specimen closer to the source to increase geometric magnification. For large magnifications, the object may thus collide with the x-ray tube during rotation, which can sometimes be circumvented by performing limited angle scans^11^. As can be seen in Fig. 1, the problem of potential collisions does not occur for CL scans, as the tilting of the rotation axis allows moving the x-ray tube’s focal spot very close to the ROI. The object itself is now allowed to have very large extents along the plane of the laminographic table. CL can therefore be considered a form of interior tomography that does not require any form of prior knowledge, if one accepts the blurring that predominantly occurs in the z-direction of the sample.

## Augmented laminography

**Figure 3:**
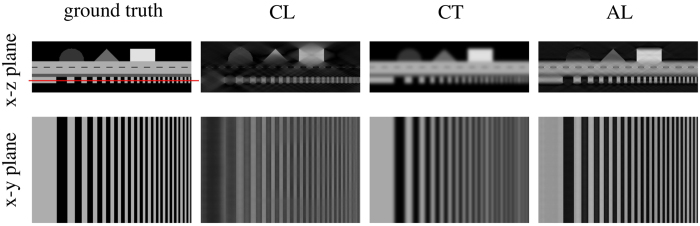
Simulations of CL, CT and AL experiments. The laminography data (*θ* = 29.8°) feature a five fold higher magnification than the CT scan. The *x*-*y* plane is indicated by the red line. The loss of resolution along the *z*-axis can be seen for the CL case, as well as an overall lower resolution exhibited by the CT scan. In the AL case, most of the CL artifacts are removed while the high resolution in the *x*-*y* plane remains.

So far, we have considered only those samples that would be handled by either CT or CL. However, there certainly exists a boundary region of specimens for which both image acquisition schemes may seem feasible. In these cases, it should be possible to combine both types of scans. Our idea was to *augment* the missing cones in the laminographic Fourier space by information obtained from a low(er) resolution CT scan, a technique we refer to as *augmented laminography* (AL). The corresponding operation is illustrated in Fig. 2, where the missing cones are partially filled with the information from a CT scan (red) of lower resolution. The part of the CT scan that augments the laminography data is marked in blue. However, still some parts are missing at high values of *k*_*z*_ due to the larger field of view and the resulting lower resolution required for the CT scan of such samples. A simulation is shown in Fig. 3 to exemplify the results of this completion of the missing data. There, a simple phantom is shown, consisting of a layered structure containing various geometric structures. The resulting CL reconstruction is shown in the center, exhibiting prominent artifacts. The CT scan is free of them, but exhibits low spatial resolution. Finally, the AL reconstruction is shown on the right. It is largely free of artifacts, but maintains a high resolution. Table 1 summarizes the key features of these three methods.

In summary, AL correlates low-resolution CT and high-resolution CL by implanting the CT information into the empty laminographic cones. In this way AL cleans coarse laminographic artifacts in the vertical planes and assures a high spatial resolution in the lateral plane of the final AL reconstruction, as illustrated in Fig. 2. Methods similar to AL, i.e. those introducing prior information from a CT scan, can especially be found in image guided radiation therapy, where the high quality planning CT can be used to remove scatter in the cone beam CT images recorded during therapy^12^,^13^,^14^. However, we are unaware of any such techniques applied to laminography.

## The study of fossils

The sample we chose to illustrate and test our approach is that of a flat fossil. Fossils are the only source of information about extinct species and help to reconstruct the origin and evolution of life. However, many fossils are preserved in sediments, which are often considerably compressed as a result of diagenetic compaction, resulting in a geometry suitable for laminography. In general, the degree of such compaction depends on the kind of matrix of the embedding sediment, as well as the timing of fossil mineralization and cementation^15^. In other words, if fossils undergo late mineralization or cementation, they will experience compression.

Due to the nature of fossils, parts of them, in particular their internal structure, are hidden. Mechanical preparation such as sectioning or serial grinding may be employed^16^,^17^,^18^, but ultimately lead to the destruction of the material. This disadvantage is also shared by methods based on electron microscopy, such as focused ion beam electron microscopy^19^ and multi-voltage back-scattered electron imaging^20^, which are additionally limited to small sample sizes, and non-intuitive to interpret. Recently, high resolution x-ray imaging became increasingly popular among palaeontologists for the non-destructive examination of fossils^21^,^22^,^23^,^24^,^25^,^26^,^27^,^28^,^29^,^30^. Both CT^31^,^32^,^33^,^34^ and CL^35^ experiments were shown to provide valuable information. Below, we demonstrate that AL can be superior to either of them by combining the best of both worlds. We do this using the example of the first fossil representative of the class Xiphosura (“horseshoe crabs”) ever found in the Mesozoic of the Netherlands^36^.

## Fossil description

**Figure 4:**
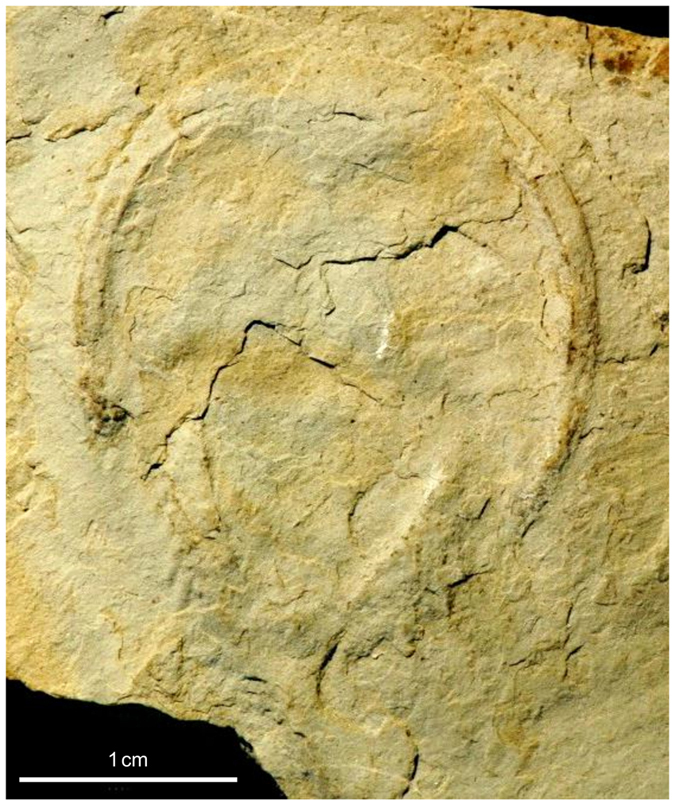
Photograph of the limulid from the Muschelkalk of Winterswijk, The Netherlands. Only the ventral side of the carapace is visible, whereas the dorsal shield of the fossil is embedded in micritic limestone.

The fossil of interest (Fig. 4) is a limulid, which belongs to the order Xiphosurida (“xiphosurids”). The xiphosurids are known to exist since the Late Ordovician^26^,^37^,^38^, i. e. at least since 445 million years ago (Mya). The taxa belonging to this group are registered in an online catalogue^39^. The specimen described here was found near the base of the Muschelkalk (Middle Triassic, Anisian) of Winterswijk, which is located in the eastern part of the Netherlands. As xiphosurids are only rarely documented in the Lower Triassic^40^,^41^,^42^, the specimen is of special interest regarding the evolution of xiphosurids. Because the carapaces of representatives of xiphosurids did not change fundamentally with time, these animals are commonly regarded as one of the best examples of “living fossils”^43^. Yet, the morphology of the carapaces is rather poor in diagnostic features, which makes it difficult to distinguish fossil taxa. Further problems to determine fossil limulids may be intraspecific variability, sexual dimorphism, differences in the morphology of different ontogenetic (instar) stages, taphonomic conditions as well as the preservation of fossils^40^. Consequently, there is still much work to be done in order to better understand fossil xiphosurid systematics and phylogenetics.

**Figure 5:**
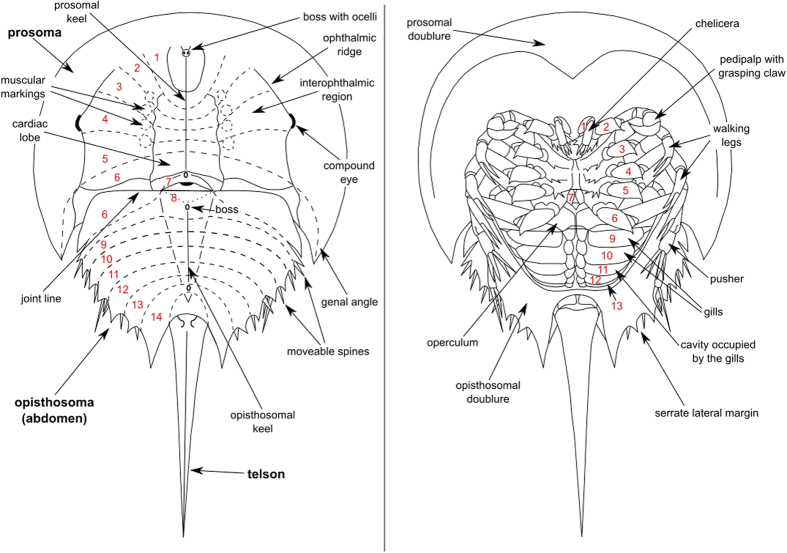
Sketch of the modern *Limulus polyphemus*, a representative of the family Limulidae. Left: Dorsal view of the carapace, which is subdivided into prosoma, opisthosoma and telson. Superimposed is the original segmentation of the carapace marked by the dashed lines. In modern taxa of xiphosurans the original segments are fused and can only be deduced from the phylogenetic development of the xiphosurans. The joint line, which subdivides the prosoma and the opisthosoma from each other, cuts the deduced course of the original 6^th^ segment. Consequently, the 6^th^ segment in modern limulids partially belongs to the prosoma and to the opisthosoma. Right: Ventral view of the carapace. The numbers 1–13 represent the appendages of the prosoma and opisthosoma. The width of the prosoma is approximately 15 cm for a male specimen.

The specimen we investigate in this work is housed in the private collection of Henk Oosterink in Winterswijk, but will later on be part of a public collection. Stratigraphically, the specimen derives from the Lower Muschelkalk (Middle Triassic, Lower Wellenkalk Member)^36^,^44^ of the still active limestone quarry south of Winterswijk. In what follows, the notation defined in the schematic of Fig. 5 is used. As parts of the specimen are of course still embedded in limestone, important diagnostic features of the prosomal shield (its carapace) such as the cardiac lobe, ophthalmic ridges with the compound eyes, joint line and genal angles remain hidden to the eye (Fig. 4). Such is also true for the operculum, which represents a cover for proximal parts of the limulid’s gills and which is of great importance for the taxonomy of xiphosurids.

Hence, only the fossil’s ventral side, i. e. its lower part, is visible. The specimen is preserved in articulation, with its prosoma, opisthosoma and part of the telson in place. Furthermore, moveable spines at the margin of the opisthosoma are also in articulation, indicating optimal taphonomic conditions such as minor or no transport and rapid burial of the specimen. The visible ventral part of the limulid comprises not much more than its outline. Only in the opisthosomal part of the carapace can the margin be distinguished from the central depression which, in the living animal, contained the gills.

The fossil is relatively small. The prosoma medially measures 16 mm in length and 21 mm including the genal angles. The maximum width of the prosoma is 25 mm. The length of the opisthosoma is estimated at approximately 15 mm, and the maximum width is also 15 mm. Only the proximal end of the telson is preserved, whereas its distal part was cut off during the recovery of the fossil. At the moment it is not possible to decide whether the specimen belongs to a small species or if it represents the juvenile stage of a larger species.

## Image Comparison

### General remarks

**Figure 6:**
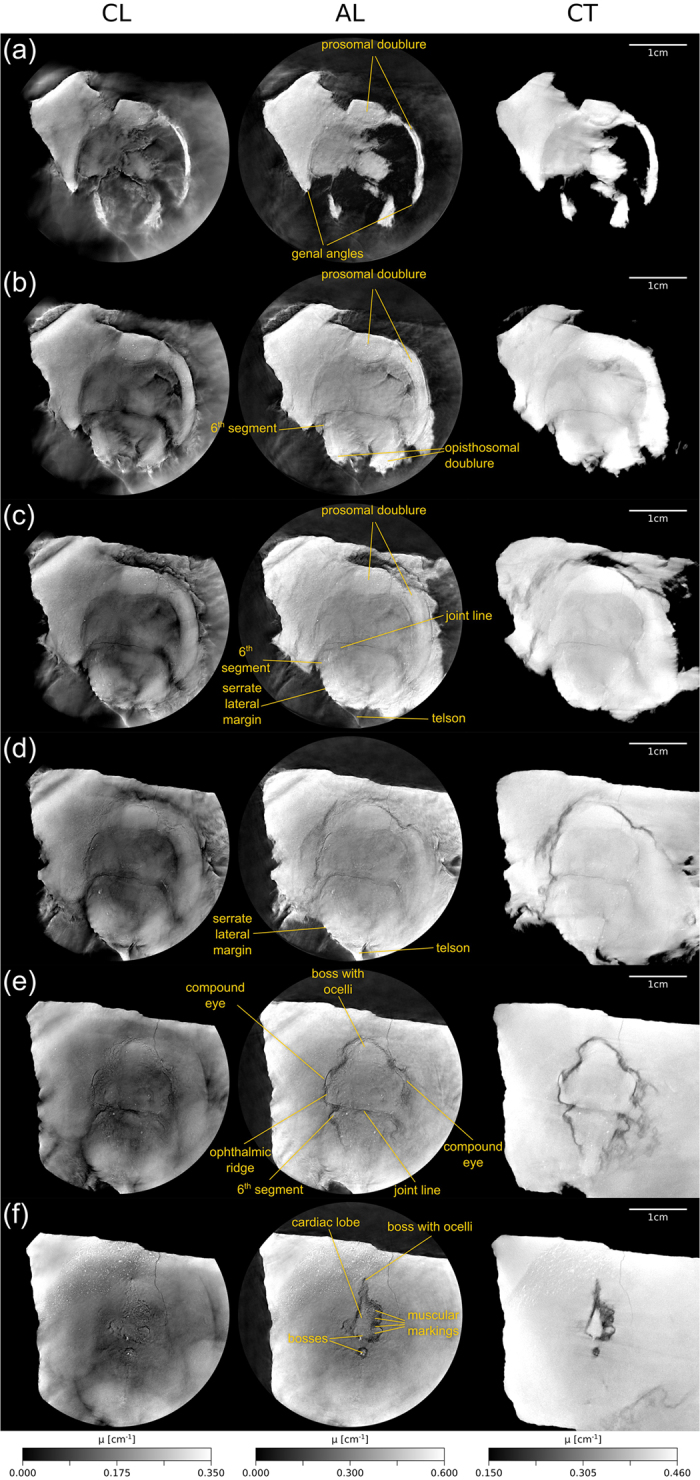
Representative slices of the limulid obtained by means of computed laminography (left), augmented laminography (middle) and computed tomography (right). The slices start at the ventral part and proceed towards the center of the specimen. The corresponding positions are marked in Fig. 7. The scale gives the values of the reconstructed attenuation coefficient *μ*.

**Figure 7:**
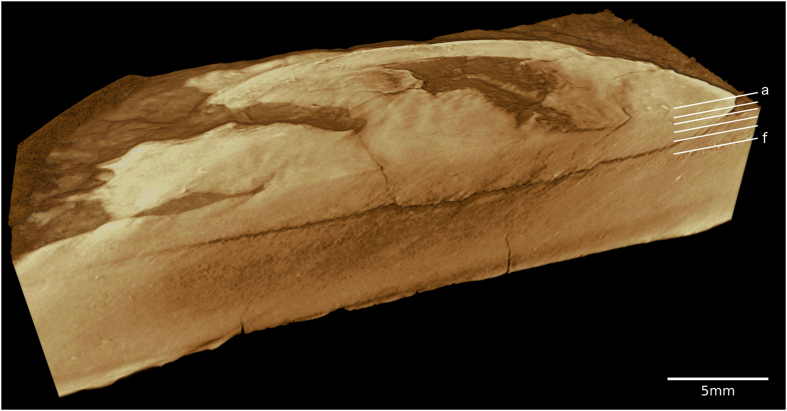
Volume rendering of the reconstructed augmented laminography volume. The positions of those slices seen in Fig. 6 are marked.

Below, we compare corresponding pairs of slices produced by all methods (CT, CL and AL), starting with the ventral part and finishing with the dorsal part of the specimen (Fig. 6). Here, the grayscale range shown was chosen individually for each method to account for the different dynamic ranges. A feature that is typical of the CL measurement is the laminographic blurring in-between slices, which gives these images a very distinct appearance. However, it can also be seen that this blurring does not occur for most of the specimen’s fine structure, which is represented by cracks in the stone and which is well resolved by laminography (especially in Fig. 6e,f). The CT slices show up in a complementary fashion. Generally, the laminographic slices resolve most high resolution features of interest, while the CT scan better visualizes the coarser outlines of structures related to the carapace. The AL images generally combine the advantages of both methods, while mostly eliminating the laminographic blurring. However, some residual artifacts are visible, which occur because we chose to perform image alignment manually in this study. Figure 7 shows a volume rendering of the AL reconstruction of the sample. Such a visualization would not be possible with the CL data.

Figure 6a: Both the CL and the CT scan show the ventral part of the carapace, in particular features of the prosoma. In both cases, the inner borders of the prosomal doublure can be traced. The same is true for the genal angles.

Figure 6b: The corresponding slices are situated slightly more dorsally than in Fig. 6a. Both the prosomal as well as the opisthosomal doublure of the carapace can be seen. The left opisthosomal portion of the 6th segment (see also Fig. 5) is also visible.

Figure 6c: These slices show similar features concerning the prosomal shield as already described in Fig. 6b. Remarkable is the broad doublure on the right side of the prosomal shield in the CL slice. It should be mentioned that the joint line, which separates the prosomal from the opisthosomal shield, can be well identified from a tiny fissure running slightly anterior in relation to the joint line. Furthermore, the opisthosomal part of the 6th segment, the joint line, the serrate lateral margin and the proximal part of the telson can be best studied in the CL slice.

Figure 6d: The CL slice here appears to provide a better visualization of details compared with the CT scan, as demonstrated in the serrate lateral margin and the telson.

Figure 6e: In these slices the dorsal features of the prosoma and opisthosoma come stronger to the fore. On the prosomal shield the ophthalmic ridge with the compound eyes can be traced best in the CT slice. The joint line is clearly visible in all slices. A boss in the frontal part of the prosoma, which can only be identified in the laminographic slice, marks the position of the median ocelli. The ocelli themselves are not visible.

Figure 6f: Here, the cardiac lobe, the muscular markings and prominent bosses on the anterior and posterior part of the prosoma are visible. While the outline of the cardiac lobe and the muscular markings can be better recognized in the CT slice, the same features show a well resolved substructure in the CL measurement.

### Detail resolution

**Figure 8:**
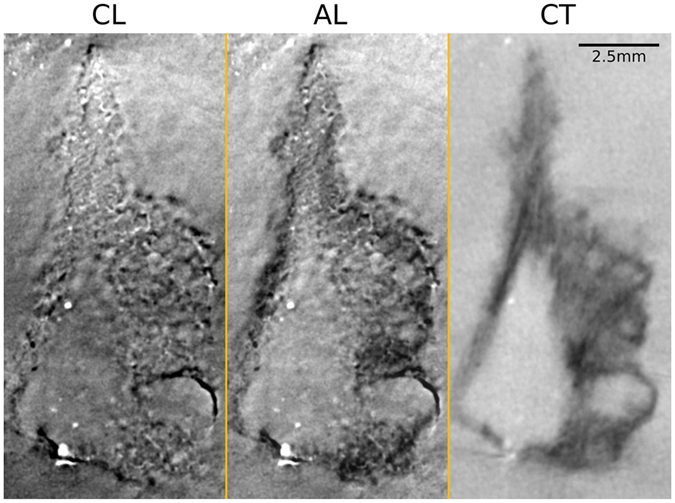
Enlarged regions taken fromFig. 6f. Image contrast was optimized individually for each method.

The slices shown in Fig. 6f demonstrate best the superior detail resolution obtainable with CL as well as the improved visualization of coarser structures by CT. To better illustrate this, Fig. 8 shows a zoomed view of the region containing the fossil. It can be clearly seen that detail resolution in the CT image is generally poor due to an excessive absorption of x-rays in the sample and a lower geometric magnification. However, the CT scan contributes coarse outlines that are masked in the laminographic images as they lie in the unsampled cones in Fourier space. The AL image thus inherits most of its sharpness from the CL scan, while broader features are obtained from the CT measurement. Without these broad features, it would be difficult to trace the fossil’s outer margins from just the CL scan.

## Fossil classification

Phylogeny and systematics of the Xiphosurida have been the subject of debate over several decades and early pre-cladistic works^45^,^46^,^47^,^48^,^49^,^50^,^51^ as well as modern phylogenetic analyses^52^,^53^,^54^,^55^ proposed different classification schemes. Recently, Lamsdell published the hitherto most comprehensive phylogenetic analysis of Xiphosurida^56^, which includes 252 characters coded for 105 taxa. Accordingly, the order Xiphosurida can be distinguished from other xiphosurans by the presence of a keel on the prosomal shield and a postabdomen, which is the narrow posterior part of opisthosoma, composed of only a single element. The Xiphosurida, however, consist of two suborders, the Palaeozoic Belinurina and the Limulina. According to Lamsdell^56^ the latter includes the genus *Bellinuroopsis*, the families Rolfeiidae and Paleolimulidae as well as the superfamily Limuloidea. The Limuloidea comprise the three taxa Limulidae, Austrolimulidae and *Valloisella*^56^. The specimen described here shows no expression of individual tergites of the opisthosoma (Fig. 6), which is a diagnostic feature of the Limulidae^56^. Furthermore, the opisthosoma of the specimen from Winterswijk is rounded and presents fixed spines (serrate lateral margin, see Fig. 6a–d), which distinguishes it from members of the clades Palaeolimulidae and Austrolimulidae. In contrast to the Limulidae, the opisthosoma of the Austrolimulidae lacks a serrate lateral margin and the posteriormost tergopleurae are swept back and elongated to form a “swallowtail”^56^. Among Middle Triassic xiphosurids the specimen from Winterswijk shows greatest affinities to the genus *Limulitella*, which is characterized by a distinct angle between the inner margin of prominent genal angles and the anterolateral margin of the opisthosoma^46^,^47^,^57^,^58^. In contrast, the genal angles of the Middle Triassic species *Tarracolimulus rieki, Heterolimulus gadeai* and *Mesolimulus crespelli* from the Muschelkalk facies of Northeastern Spain are less prominent^59^. Furthermore, the prosoma of *Limulitella* is broader than the opisthosoma^46^, which also distinguishes the genus from *Heterolimulus gadeai*^60^ and *Mesolimulus crespelli*^59^. It should also be mentioned that the specimen from Winterswijk resembles the species “*Limulitella*” *henkeli* from the Muschelkalk of Saxony Anhalt, Germany, with respect to the course of the joint line, the development of the genal angles and the dimensions of the prosoma and opisthosoma. However, the preservation of this specimen does not allow a precise comparison. According to Lamsdell^56^ the genus *Limulitella* is polyphyletic. Due to the existing taxonomic uncertainties, a revision is necessary. *Yunnanolimulus luopingensis* from the Muschelkalk facies of Southern China^61^ has a similar shape of the prosoma and opisthosoma, but differs from the Winterswijk specimen by the presence of lateral ridges on the opisthosoma, which bear a row of prominent pits. As a result, the assignment of the specimen to the genus *Limulitella*, which had been supposed earlier^36^, can be confirmed now by the results produced by AL. As shown in Fig. 4, the joint line is not visible in the original specimen, because it is covered with a thin layer of sediment. Now, it is possible to reconstruct the shape of the carapace including the joint line and its relationship to the genal angles by means of AL. Moreover, the dorsal side of the carapace can be visualized.

## Conclusions

The aim of the present work was to compare the two methods computed tomography (CT) and computed laminography (CL) with each other to analyze a relatively flat and extended fossil. We found that it is not possible to generally favor a single one of these techniques for the investigation of such a sample. On the contrary, each method provides specific information about the object investigated. Generally, the CT images are characterized by a coarser, yet constant resolution in each direction, while CL images trade out-of-plane resolution for an in-plane sharpness. Whereas CL images are generally rich in contrast, the contrast of parts of the CT scan is relatively low (Fig. 6a–d) and affected by image noise arising from long photon penetration depths. Consequently, a remarkable difference between the two methods is that those fossil structures that are revealed in the CL scan are highly resolved. However, the CT images better visualize coarser outlines of particular structures (Figs 6e,f and 8). These structures will be hidden in the CL measurement if the corresponding spatial frequencies lie within the two cones in Fourier space that remain unsampled (Fig. 2).

The complementary nature of CT and CL led us to propose their combination in Fourier space, a method we introduced as augmented laminography (AL). As shown in the central column of Fig. 6, AL provides a larger amount of information for most structures. In the future, we believe fine tuning the weighting of the individual acquisition methods prior to their combination could further improve detail recognition.

AL allowed us to derive important conclusions about the fossil studied and thus to classify it as the genus *Limulitella*. The new results now facilitate a comparison between the specimen described here and all known specimens of this particular genus.

## Methods

**Figure 9:**
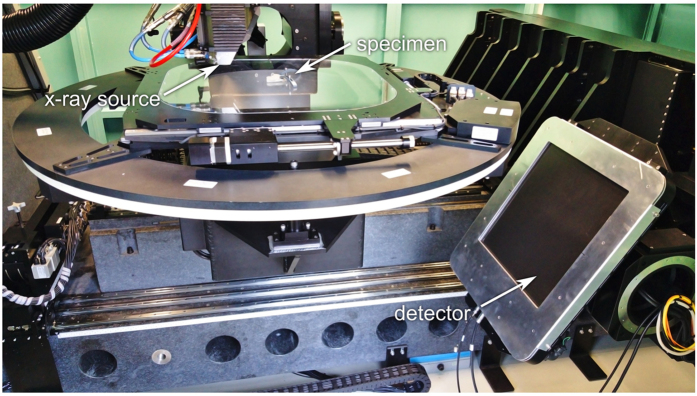
Photograph of the laminography configuration. While the axis of rotation is perpendicular, both x-ray tube and detector are in an inclined orientation, facilitating the laminographic angle of around 29.8°. The distance between source and detector is 1135 mm.

Mechanical preparation was precluded due to the fragility and rareness of the fossil. X-ray analysis was carried out in the tomography lab at the ANKA synchrotron. The scanner hosted therein is based on a custom design making use of a microfocus x-ray tube and a flat panel detector (Fig. 9). It was specifically designed to enable both CT and CL acquisitions in a cone beam geometry. A microfocus x-ray tube (XWT-225, X-RAY WorX, Garbsen, Germany) was employed and operated with a flat panel detector (XRD 1621 CN14 ES, PerkinElmer, Waltham, USA), featuring a pixel size of 200 *μ*m and a DRZ+ scintillator. The effective voxel size in the resulting image stacks is therefore 200 *μ*m/*M*. Here, *M* = SDD/SOD is the magnification factor that derives from the source-to-detector-distance (SDD) and the source-to-object distance (SOD).

In order to mitigate beam hardening artifacts, we employed a comparably high tube voltage of 200 kV, and pre-hardened the resulting x-ray spectrum by a 1 mm thick copper filter. Since our laminography setup required penetrating additional 3 mm of aluminium, we added the same amount also to the CT scan to achieve comparable results. We then recorded 2048 projections over a total angular interval of 360°. The acquisition time per projection was 64 s, resulting in a total scanning time of 36.4 h (38.8 h including detector read-out and step-wise rotation).

The CL scan was performed equivalently to the CT case. In particular, we used the same value of 1135 mm for the SDD to enable a fair comparison. The SOD was 220 mm in the CT case, amounting to a magnification of *M* = 5.16. However, to exploit the higher magnification possible with laminography, we reduced the SOD to a value of 137 mm during the CL scan, which corresponds to a magnification of *M* = 8.28. The laminographic angle was approximately 29.8°. Instead of tilting the object, which would have been an option with our scanner, we chose to tilt both source and detector, while leaving the object table in a horizontal orientation. We did this to avoid a possible failure of the clamps pressing the fossil onto the plexiglass substrate during the long scan. Such a failure would have made the fossil slide from the object table when in a non-horizontal configuration.

In addition to the projection images of the specimen, flat field images (for homogeneity corrections) and acquisitions without x-rays (to subtract the detector’s dark current) were recorded. During flat field acquisitions, the sample was moved out of the field of view using an open frame XY-stage. The aluminium cone beneath it remained stationary during this procedure. Reconstructions for both CT and CL were performed using the software Octopus 8.6 (Inside Matters, Gent, Belgium). For AL, both reconstructed volumes were aligned manually and resampled to exhibit equal image dimensions. The missing parts in the Fourier space of the CL scan were identified by comparing the amplitude of the 3D Fourier transform of both volumes and applying a threshold. The resulting binary mask was median filtered and used to select the Fourier coefficients from the CT measurement to fill the empty cones in the CL scan. The final AL 3D image volume was then obtained by means of an inverse Fourier transform.

## Additional Information

**How to cite this article**: Zuber, M. *et al*. Augmented laminography, a correlative 3D imaging method for revealing the inner structure of compressed fossils. *Sci. Rep.* **7**, 41413; doi: 10.1038/srep41413 (2017).

**Publisher's note:** Springer Nature remains neutral with regard to jurisdictional claims in published maps and institutional affiliations.

## Figures and Tables

**Table 1:** Key features of CL, AL, and CT.

CL	AL	CT
+High resolution in *xy* plane.	+High resolution in *xy* plane.	+Uniform, but potentially reduced resolution in *x, y, z*.
−Blurring in *z* direction.	+Medium resolution in *z* direction, i.e. typical CL artifacts are suppressed to a large degree.	−Sample has to fit into the FOV.
+Large geometric magnifications can be achieved.	−Increased scanning times.	−Strong attenuation for flat, extended objects.
+Reduced attenuation for flat, extended objects.		
